# Flavonoids from *Ficus pandurate* var. *angustifolia* W.C. Cheng Restore Cognitive Impairment and Regulate the Gut Microbiota in Sleep-Deprived Mice

**DOI:** 10.3390/foods14162888

**Published:** 2025-08-20

**Authors:** Haochen Dai, Songmei Luo, Xin Zhang

**Affiliations:** 1 Department of Food Science and Engineering, Ningbo University, Ningbo 315211, China; 2311390108@nbu.edu.cn; 2 Department of Pharmacy, Wenzhou Medical University, Lishui 323000, China

**Keywords:** *Ficus pandurata* var. *angustifolia* W.C. Cheng, flavonoids, gut microbiota, inflammation, cognition

## Abstract

Sleep deprivation (SD) induces cognitive impairment associated with gut microbiota dysbiosis, making it crucial to explore natural remedies targeting the microbiota–gut–brain axis. This study aims to investigate whether *Ficus pandurata* var. *angustifolia* W.C. Cheng (a traditional medicine–food plant rich in flavonoids) can mitigate cognitive impairment caused by SD by modulating the gut microbiota. The sleep-deprived mouse model was established using the multiple platform water environment method. This study investigated the effects of *F. pandurata* var. *angustifolia* flavonoids (FCFs) via behavioral tests, 16S rRNA sequencing, and biochemical analyses to assess cognitive function, gut microbiota, and related pathways. FCF alleviated SD-induced cognitive deficits, reversed gut microbiota dysbiosis (increased beneficial bacteria like *Lactobacillus*, reduced harmful ones like *Desulfovibrio*), promoted short-chain fatty acids production, improved colonic histopathology and intestinal barrier function, reduced serum lipopolysaccharide, inhibited glial cell activation and TLR4/NF-κB signaling, and regulated neurotransmitters. In conclusion, FCF ameliorates SD-induced cognitive impairment through regulating gut microbiota, enhancing intestinal barrier, and suppressing neuroinflammation via the microbiota–gut–brain axis, providing a theoretical basis for its application.

## 1. Introduction

*Ficus pandurate* var. *angustifolia* W.C. Cheng is a small deciduous shrub belonging to the *Ficus* genus of the Moraceae family, widely distributed in subtropical regions of China, particularly in Zhejiang Province and other adjacent areas [1]. Traditionally used as a medicine–food homologous plant by the She ethnic group, its roots, stems, and leaves have dual applications: as herbal medicine for improving blood circulation, reducing inflammation, and alleviating pain, and as a seasoning in cooking [2]. With advances in artificial cultivation and regional policy support, large-scale cultivation of *F. pandurata* var. *angustifolia* has been promoted, spurring growing research interest in its active components and applications [3,4]. In the field of natural product research, flavonoids have attracted significant interest due to their multi-target anti-inflammatory and neuroprotective properties [5,6]. Preliminary studies have shown that *F. pandurata* var. *angustifolia* flavonoids (FCFs) can regulate mouse gut microbiota, inhibit proinflammatory cytokine expression in vivo, and exert neuroprotective effects [7].

Sleep, as a core physiological process maintaining mammalian homeostasis, is inherently important [8]. Sleep deprivation (SD) refers to the inability to meet the physiological sleep requirements due to personal or environmental factors, leading to various functional disorders in the endocrine, metabolic, cardiovascular, and nervous systems [9]. However, in modern society, SD has become an increasingly common threat to public health. Although currently clinically used sedative–hypnotic drugs, such as benzodiazepines and non-benzodiazepine hypnotics, can relieve sleep disturbances, their long-term use is limited by notable drawbacks [10,11]. Benzodiazepines often induce tolerance and physical dependence, leading to dose escalation and difficult withdrawal, while non-benzodiazepines may cause residual sedation, memory impairment, and rebound insomnia [12]. These adverse effects affect patients’ quality of life and increase the risk of long-term neurological complications. Therefore, actively exploring safe and effective non-pharmacological intervention strategies is of great significance [13].

The gut microbiota, also termed the “second brain”, communicates bidirectionally with the central nervous system (CNS) via immune, endocrine, and metabolic signaling pathways [14]. The gut–brain axis is a bidirectional interaction network involving the CNS, autonomic nervous system, enteric nervous system, immune system, and endocrine system. Its core function lies in coordinating the cognitive regulation of the CNS with peripheral intestinal physiology, and achieving co-monitoring and precise regulation of intestinal homeostasis and brain cognitive activities through signal transmission among multiple systems [15]. Mounting evidence indicates that the gut microbiota modulates intestinal barrier integrity and intestinal inflammatory responses, while also regulating glial cell activation by generating metabolites such as short-chain fatty acids (SCFAs) and neurotransmitters [16,17].

SD induced by external environmental factors may result in abnormal intestinal permeability and gut microbiota dysbiosis [18]. The pathophysiological process of SD has been confirmed to be intimately linked to gut microbiota dysbiosis [19]. Experimental evidence shows that transferring the gut microbiota from SD mice to germ-free mice can trigger elevated levels of hippocampal inflammatory factors, activation of microglia, and cognitive impairment in recipients, confirming that gut microbial dysbiosis exerts a causal effect on cognitive impairments related to SD [20]. Gut microbiota disorders can decrease intestinal mucosal tight junction proteins, increase intestinal barrier permeability, enable lipopolysaccharide (LPS) to enter the bloodstream, and trigger systemic inflammation, thereby weakening the blood–brain barrier (BBB) function. LPS binds to Toll-like receptor 4 (TLR4) on microglia, activating proinflammatory pathways, ultimately leading to inflammatory damage to hippocampal neurons [21,22].

Flavonoids can regulate the gut microbiota, and changes in the gut microbiota affect neural function through the microbiota–gut–brain axis. Therefore, this study aimed to investigate the effects of FCF on the gut microbiota and cognitive function in sleep-deprived mice, with a focus on elucidating whether and how FCF alleviates sleep deprivation-induced cognitive impairment through the microbiota–gut–brain axis. Our findings may offer novel insights into SD and support the development of therapeutic strategies for SD-associated cognitive impairment.

## 2. Materials and Methods

### 2.1. Materials and Reagents

*F. pandurata* var. *angustifolia* was collected from Lishui City, Zhejiang Province, China. Melatonin (cat. no. S20287) and sodium carboxymethyl cellulose (cat. no. S14017) were purchased from Shanghai Yuanye Biotechnology Co., Ltd. (Shanghai, China). The experimental mice came from Beijing Vital River Laboratory Animal Technology Co., Ltd. (Beijing, China). Ningbo Experimental Animal Center provided the ordinary animal feed. Detection ELISA kits for interleukin-1β (IL-1β, cat. no. KX2040), tumor necrosis factor-α (TNF-α, cat. no. KX2132), interleukin-6 (IL-6, cat. no. KX2163), LPS (cat. no. KX2631), 5-hydroxytryptamine (5-HT, cat. no. KX2443), γ-aminobutyric acid (GABA, cat. no. KX3554), and glutamate (GLU, cat. no. KX3014) were purchased from Shanghai Kexing Biotechnology Co., Ltd. (Shanghai, China).

### 2.2. Preparation of FCF

The extraction and purification of FCF were performed following our laboratory’s previously established protocols [7]. Briefly, a suitable amount of dried root powder of *F. pandurata* var. *angustifolia* was added to 70% ethanol at a solid-to-solvent ratio of 1:40 (*w*/*v*). The mixture underwent ultrasonic-assisted extraction for 60 min, after which the extract was centrifuged to obtain the supernatant. The residue was re-extracted using the same protocol. The supernatants from both extractions were combined, adsorbed onto a polyamide column, and eluted with an 80% ethanol solution. Following column processing, the filtrate was concentrated via rotary evaporation and lyophilized to yield the FCF sample, which was stored at −20 °C for subsequent experiments. Post-purification, the total flavonoid content was quantified using rutin as a standard curve.

### 2.3. Analysis of the Components of FCF

Compositional analysis of FCF was conducted based on previously reported protocols, with slight modifications [23]. FCF compounds were analyzed using an UltiMate 3000 UPLC system coupled with a TripleTOF 6600 mass spectrometer (SCIEX, Redwood City, CA, USA). Chromatographic separation was achieved on a Poroshell 120 EC-C18 column (2.1 × 100 mm, 2.7 µm) maintained at 40 °C with an injection volume of 4 μL. The mobile phase consisted of (A) 5 mmol/L ammonium formate in water containing 0.1% formic acid and (B) 5 mmol/L ammonium formate in 95% acetonitrile containing 0.1% formic acid. The mass spectrometer operated in multiple reaction monitoring (MRM) mode with an electrospray ionization (ESI) source. The ion source temperature was 550 °C, with positive and negative ion modes, spray voltages of +4500 V and −4500 V, respectively, and curtain gas (CUR) pressure of 25 psi.

### 2.4. Animal Experiment Design

Male SPF-grade C57BL/6J mice (6–8 weeks old, 22 ± 2 g) were purchased from Beijing Vital River Laboratory Animal Technology Co., Ltd. and housed in the SPF barrier system of Ningbo University Experimental Animal Center under controlled conditions (temperature: 22 ± 1 °C, humidity: 55 ± 5%). Mice were acclimated to a 12/12 h light/dark cycle for 1 week. After acclimation, mice were randomly divided into four groups (15 mice per group): the control (CT) group, the sleep deprivation (SD) group, the sleep deprivation + FCF (FCF) group, and the sleep deprivation + melatonin (MT) group. The CT and SD groups received 0.2 mL of 0.5% carboxymethyl cellulose sodium (CMC-Na), while FCF and melatonin were suspended in 0.5% CMC-Na for gavage. The FCF group received 0.2 mL of FCF (100 mg/kg BW), and the MT group received 0.2 mL of melatonin (50 mg/kg BW), administered continuously for 3 weeks. Gavage was performed before daily SD. Body weight was recorded weekly.

The SD, FCF, and MT groups underwent SD from 16:00 to 10:00 daily (18 h/day) for three consecutive weeks (21 days) using the modified multiple platform water environment method (MMPM) [24]. The bottom of the sleep deprivation tank was evenly fitted with 20 cylindrical platforms (2.5 cm diameter, 7 cm height) spaced 5 cm apart. Water was added to maintain a level 1 cm below the platform surface, with cleanliness checked and maintained multiple times daily. Food and water were positioned 3 cm above the platforms for free access. No more than ten mice were housed per tank, acclimated to the platforms for 2 days (1 h/day) prior to the experiment, followed by 18 h/day SD for 3 weeks. SD was achieved as mice involuntarily awoke due to head-dropping into water or falling off platforms caused by muscle relaxation during sleep. The study protocol was designed to minimize animal numbers and suffering and was approved by the Laboratory Animal Care Ethics Committee of Ningbo University (permission number: SYXK [Zhejiang] 2019-0001).

### 2.5. Experimental Sample Preparation

Fecal samples were gathered using sterile cryopreservation tubes and promptly stored at −80 °C for fecal microbiota analysis and SCFAs analysis. Following behavioral tests, mice were euthanized with tribromoethanol, and serum was collected from the orbital plexus. After intracardiac perfusion, the colon and hippocampus tissues were fixed with 4% paraformaldehyde or stored at −80 °C in a freezer for subsequent assays.

### 2.6. Behavioral Assessment

Spatial learning and memory were evaluated using the Morris water maze test, following procedures described in earlier studies [25]. Spatial learning and memory were assessed with the Morris water maze. During place navigation (days 1–5, two trials daily), trajectories and escape latency within 60 s were recorded, and animals that failed to locate the platform within 60 s were assigned the maximum latency of 60 s. Spatial exploration on day 6 (platform removed) quantified platform crossings and the percentage of time spent in the target quadrant within 60 s.

Anxiety-like behavior was assessed using the open-field test, based on protocols described previously [26]. The open-field test evaluated these parameters by placing mice in the center of a white chamber and recording total movement distance, distance traveled in the central zone, and time spent in the central zone during a 5 min session.

Hippocampus-dependent recognition memory was evaluated using the novel object recognition (NOR) test, with reference to earlier procedures [27]. In the NOR test, mice explored two identical objects for 10 min on day 1, and on day 2, one object was replaced, and 5 min exploration times for both objects were recorded. The recognition index (RI) was calculated as (exploration time of novel object)/(exploration time of novel object + exploration time of old object) × 100%.

### 2.7. Gut Microbiota Analysis

Fecal microbial DNA was extracted with the Fecal Genome DNA Extraction Kit (BioTeke, Beijing, China) and quantified using Qubit (Invitrogen, Carlsbad, CA, USA). PCR amplification of the 16S rRNA gene was performed with primers 341F/805R under specific conditions, and the product was purified and quantified. Qualified products were sequenced on an Illumina NovaSeq 6000 (Illumina, San Diego, CA, USA). Data processing included removing sequencing primers, merging paired-end reads, trimming low-quality reads, and filtering chimeric sequences. DADA2 denoised and generated ASVs, with sequence alignment via QIIME2 feature-classifier using SILVA and NT-16S databases [28]. Alpha and beta diversities were calculated via QIIME2. Other diagrams were made with the R package 3.4.4 [29].

### 2.8. SCFA Extraction and Analysis

The concentrations of SCFAs in fecal samples were determined following previously described methods [30,31]. Accurately weigh 50 mg of sample, add 1000 μL of −80 °C pre-cooled 80% methanol water to precipitate protein, perform steel ball milling and vortex extraction for 20 min, centrifuge at 20,000× *g* and 4 °C for 15 min, transfer 20 μL of supernatant to a 1.5 mL EP tube, add EDC solution and 3-NPH for derivatization, add initial mobile phase solution to 500 μL, vortex to mix, and use the supernatant for LC-MS/MS analysis. UPLC conditions: Separate and quantify target compounds on an AB Sciex JasperTM ultra-performance liquid chromatograph (SCIEX, Framingham, MA, USA) coupled with an AB SCIEX 4500MD triple quadrupole mass spectrometer (SCIEX, Framingham, MA, USA) using an Agilent Poroshell 120 EC-C18 2.7 μm (2.1 × 100 mm) column (Agilent Technologies, Santa Clara, CA, USA), with mobile phases of pure water (A) and methanol + acetonitrile (*v*/*v* = 1:1, B), injection volume of 1 μL, and column temperature of 40 °C. ESI-MS/MS operates in negative ion mode with a turbo spray ion source at 400 °C and an ion spray voltage of −3000 V.

### 2.9. Histological Examination

Histological examination of colon and brain tissues was performed following previously described methods with minor modifications [32]. Colon and brain were fixed with 4% paraformaldehyde, then paraffin-embedded and sectioned into 5 μm slices. Sections were deparaffinized with xylene, rehydrated through graded ethanol (70–95%), washed with PBS (pH 7.4), stained with H&E, and examined under a microscope (Motic, Xiamen, China).

### 2.10. Biochemical Measurement

A specific amount of hippocampal tissue was homogenized, and the homogenate was centrifuged to obtain the supernatant. Serum samples were directly used. According to the kit instructions, the LPS content in serum, the levels of inflammatory cytokines IL-6, IL-1β, and TNF-α in both hippocampal supernatant and serum, as well as the neurotransmitters GABA, GLU, and 5-HT in the hippocampal supernatant, were determined by ELISA.

### 2.11. Western Blot

Western blotting analysis was performed following procedures described in previous studies [33]. The total protein concentration was determined using a bicinchoninic acid protein assay kit. Equal amounts of protein samples from the supernatants were separated by sodium dodecyl sulfate–polyacrylamide gel electrophoresis and transferred to polyvinylidene fluoride membranes. After transfer, the membranes were blocked with 5% bovine serum albumin for 2 h. Diluted primary antibodies, including TLR4 antibody (1:1000, Proteintech, Rosemont, IL, USA, cat no. 19811-1-AP), p-NF-κB p65 antibody (1:1000, Cell Signaling Technology, Danvers, MA, USA, cat no. 3033T), NF-κB p65 antibody (1:1000, Cell Signaling Technology, cat no. 8242T), and β-actin antibody (1:5000, Proteintech, cat no. 66009-1-Ig), were added and incubated overnight at 4 °C. Subsequently, the membranes were incubated with horseradish peroxidase-conjugated goat anti-mouse/rabbit secondary antibodies (1:8000, Proteintech, cat no. SA00001-1) at room temperature for 2 h, washed with Tris-buffered saline with Tween 20, and developed using a chemiluminescent substrate. Protein bands were semi-quantitatively analyzed using ImageJ 1.54g software.

### 2.12. Immunofluorescence Analysis

Immunofluorescence analysis was performed following previously described methods with minor modifications [34]. Tissue sections were dried at 37 °C, fixed with 4% paraformaldehyde, air-dried, washed with PBS, subjected to EDTA-based antigen retrieval, outlined with a blocking pen, and blocked to prevent non-specific binding. They were incubated with primary antibodies overnight at 4 °C, washed three times with PBS, and then incubated with secondary antibodies in the dark. Nuclei were stained with DAPI, autofluorescence was quenched, slides were mounted, and glial cell fluorescence intensity was observed under a fluorescence microscope and analyzed with ImageJ.

### 2.13. Immunohistochemistry Analysis

Immunohistochemistry analysis was carried out in accordance with methods described previously, with slight modifications [35]. Tissue sections underwent antigen retrieval with citrate buffer via microwave heating, blocked for endogenous peroxidase in the dark at room temperature, and then blocked with serum for 30 min. After overnight incubation with PBS-diluted primary antibodies at 4 °C and thorough PBS washes, sections were incubated with HRP-conjugated secondary antibodies for 50 min at room temperature. Chromogenic development was performed with diaminobenzidine, and nuclei were counterstained with hematoxylin. Sections were dehydrated through graded ethanol, cleared, mounted, imaged under a light microscope, and quantified with ImageJ.

### 2.14. Statistical Analysis

Data obtained were analyzed using SPSS 23 software, with results expressed as mean ± standard deviation from at least three replicates. Data plotting was performed using GraphPad Prism 10.1.2. Statistical significance between groups was evaluated by one-way analysis of variance (ANOVA) followed by Dunnett’s multiple comparison test (* *p* < 0.05, ** *p* < 0.01, *** *p* < 0.001).

## 3. Results

### 3.1. Analysis of FCF Components

The total flavonoid content of FCF was determined as 78.49%. Compositional analysis of FCF is presented in Table 1, with representative MRM spectra and chemical structures of the flavonoids shown in Figure S1. Quantitative profiling revealed a diverse flavonoid composition, with genistein and apigenin as the most abundant components, followed by luteolin, rutin, cynaroside, and combined isoquercitrin and hyperoside in descending order of content.

### 3.2. Effect of FCF on Body Weight and Spatial Memory in SD Mice

**Figure 1:**
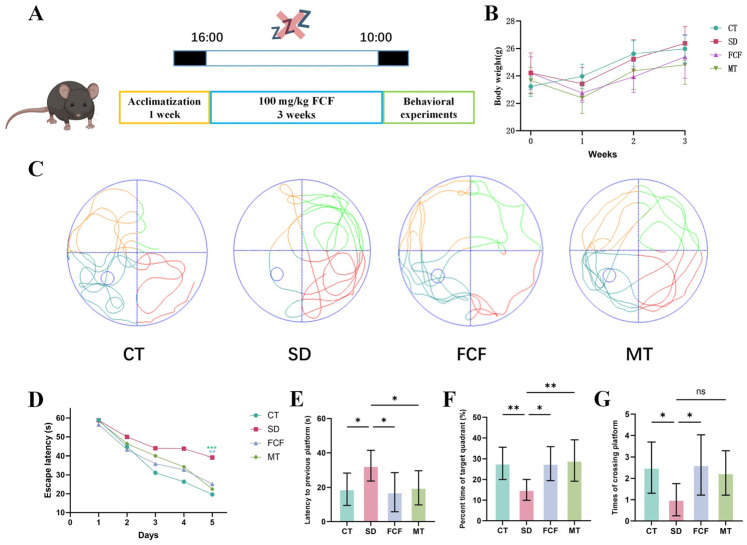
Effect of FCF on body weight and spatial memory in sleep-deprived mice. (**A**) Schematic diagram of the experimental procedures. (**B**) Changes in body weight during the 3-week experiment interval. (**C**) Spatial memory track diagram in Morris water maze test (no hidden platform). (**D**) Escape latency during training sessions. (**E**) Escape latency during testing sessions. (**F**) Percentage of time spent in the target quadrant. (**G**) Number of platform crossings in the target quadrant. Data are presented as mean ± standard deviation (*n* = 8). * *p* < 0.05, ** *p* < 0.01, *** *p* < 0.001, ns: no significant difference vs. the SD group. Statistical analysis was performed using one-way ANOVA.

To investigate whether FCF treatment alleviates sleep deprivation-induced cognitive impairment, sleep-deprived mice were administered with FCF (100 mg/kg BW) for 3 weeks (Figure 1A). After 3 weeks of feeding with a standard diet, it was observed that the body weight of the SD group showed a slight decrease in the first week of SD, followed by a more significant increase over the subsequent two weeks. FCF treatment reduced the rate of body weight gain in mice (Figure 1B). These findings indicate that FCF can attenuate SD-induced body weight gain. Compared to the FCF group, the MT group was more effective in reducing SD-induced body weight gain. However, both groups showed no significant difference compared to the SD group.

The Morris water maze was used to assess hippocampal spatial learning and memory in mice. In the water maze experiment, trajectory plots (Figure 1C) revealed disorganized paths in the SD group, with markedly reduced activity in the target quadrant. Mice in the FCF and MT groups demonstrated significantly increased movement in the target quadrant. During the training phase, escape latency—the time taken for mice to locate the platform—gradually decreased, whereas SD mice showed a slower rate of reduction compared to the other three groups (Figure 1D). In the test phase, SD mice exhibited significantly longer escape latencies than CT, FCF, and MT groups (*p* < 0.01, *p* < 0.05, *p* < 0.01) (Figure 1E), indicating impaired spatial learning and memory. Compared to the SD group, FCF-treated mice spent a significantly higher proportion of time in the target zone (*p* < 0.01) and showed more platform crossings (*p* < 0.05) during the test phase (Figure 1F,G). Moreover, the MT group spent a higher proportion of time in the target zone than the FCF group, but exhibited no significant difference in the number of platform crossings compared to the SD group (Figure 1F,G).

### 3.3. Effect of FCF on Anxiety-like Behavior and Recognition Memory in SD Mice

**Figure 2:**
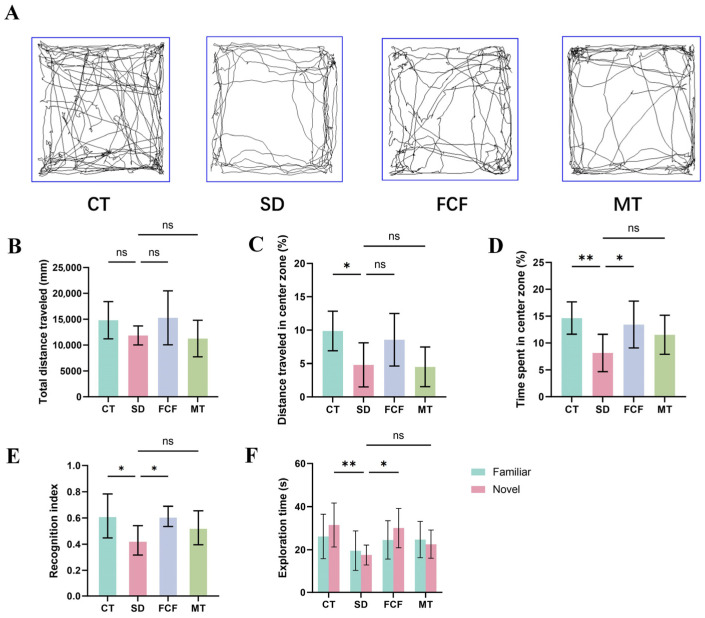
Effect of FCF on anxiety-like behavior and recognition memory in sleep-deprived mice. (**A**) Representative movement trajectories in the open-field test. (**B**–**D**) Total movement distance, percentage of movement distance in the central area, and percentage of residence time in the central area of mice in the open-field test. (**E**,**F**) RI and exploration time for novel vs. familiar objects in the novel object recognition test. Data are presented as mean ± standard deviation (*n* = 8). * *p* < 0.05, ** *p* < 0.01, ns: no significant difference vs. the SD group. Statistical analysis was performed using one-way ANOVA.

The open-field test was employed to assess anxiety-like behavior and locomotor activity in mice. Representative trajectory plots from the open-field test (Figure 2A) showed that CT, FCF, and MT groups preferred to navigate the central area compared to the SD group. Statistical analysis revealed no significant differences in total movement distance among the SD, FCF, and MT groups relative to the CT group (Figure 2B). The SD group exhibited significantly reduced distance traveled and time spent in the central area compared to CT mice (*p* < 0.05, *p* < 0.01) (Figure 2C,D), whereas FCF treatment significantly increased the proportion of time spent in the central area (*p* < 0.05) (Figure 2D), indicating enhanced willingness to explore central regions.

Additionally, the NOR test was conducted to evaluate hippocampus-dependent recognition memory. Compared to CT mice, the SD group showed a significantly lower RI (*p* < 0.05) and drastically reduced exploration time for the novel object (*p* < 0.01) (Figure 2E,F), indicating impaired short-term memory and diminished performance due to SD. The MT group performed poorly in this test, showing no significant differences from the SD group in terms of RI and novel object exploration time (Figure 2E,F). FCF treatment significantly improved RI and novel object exploration time in SD mice (*p* < 0.05).

### 3.4. Effect of FCF on the Gut Microbiota and SCFAs of SD Mice

**Figure 3:**
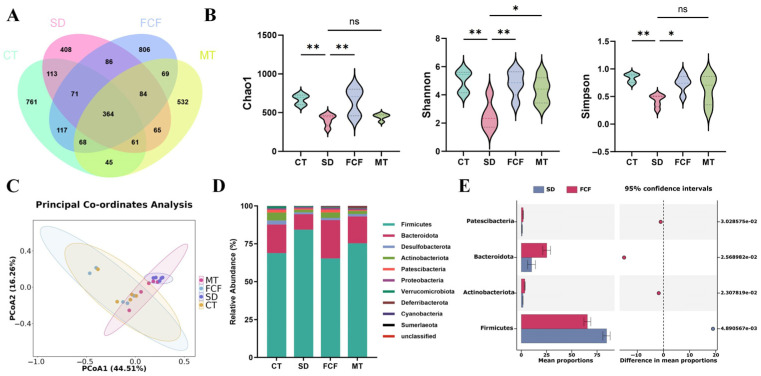
Effects of FCF on diversity and phylum-level changes in gut microbiota in sleep-deprived mice. (**A**) Venn diagram of gut microbial OTUs across four mouse groups. (**B**) α-diversity analysis: effects on Chao1, Shannon, and Simpson indices. (**C**) β-diversity analysis: PCoA based on Bray–Curtis distance metric. (**D**) Relative abundances of gut microbiota at the phylum level across four groups. (**E**) Significantly differentially abundant phyla between the SD and FCF groups. Data are presented as mean ± standard deviation (*n* = 5). * *p* < 0.05, ** *p* < 0.01, ns: no significant difference vs. the SD group. Statistical analysis was performed using one-way ANOVA.

To fully understand how FCF treatment alters microbiota composition, 16S rDNA amplicon sequencing was performed on fecal samples from each group of mice. A Venn diagram (Figure 3A) was used to calculate the mean and overlap of OUTs across groups, outlining the effects of five experimental treatments on gut microbiota abundance. The four groups shared 364 OUTs. The CT, SD, FCF, and MT groups had 761, 408, 806, and 532 specific OUTs, respectively. Gut microbiota alpha diversity, reflecting community richness, was evaluated using indices such as Chao1, Shannon, and Simpson (Figure 3B). Compared with the CT group, the SD group showed significantly reduced Chao1, Shannon, and Simpson indices (*p* < 0.01), indicating that SD decreased microbial community diversity. FCF intake largely restored gut microbiota alpha diversity in mice (*p* < 0.01, *p* < 0.01, *p* < 0.05). In contrast, MT treatment increased all three indices, but only the Shannon index showed a significant difference (*p* < 0.05). To measure the similarity between microbial communities, beta diversity (PCoA) was further assessed to analyze species differences among the four groups (Figure 3C). The microbial composition of the SD group was distinct from that of the CT group, indicating intergroup differences in gut microbiota. However, FCF treatment increased gut microbiota diversity and richness and reversed the community differences caused by SD.

At the phylum level, the main gut bacterial phyla were *Bacteroidota*, *Firmicutes*, *Desulfobacterota*, and *Actinobacteriota* (Figure 3D). The abundance of *Actinobacteriota* microbiota decreased after 3 weeks of SD but was reversed by FCF treatment (Figure 3E). The *Firmicutes*/*Bacteroidota* (F/B) ratio in the SD group was significantly higher than that in the FCF group. After FCF feeding, the abundance of *Firmicutes* (F) decreased, while that of *Bacteroidota* (B) increased.

**Figure 4:**
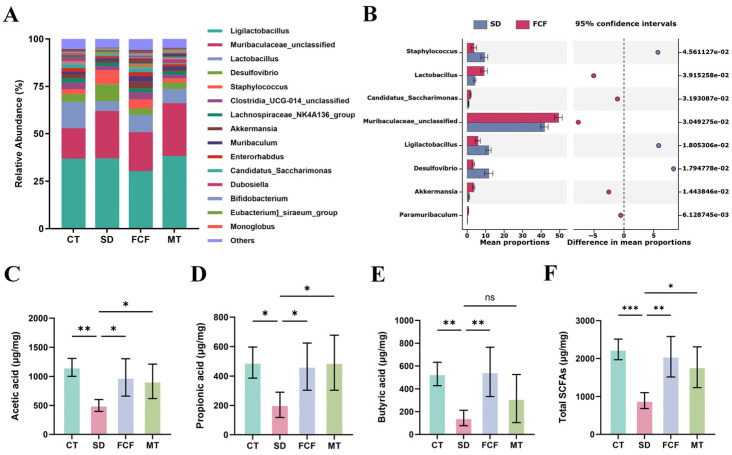
Effects of FCF on genus-level changes in gut microbiota and SCFA levels in sleep-deprived mice. (**A**) Relative abundances of gut microbiota at the genus level across four groups. (**B**) Significantly differentially abundant genera between the SD and FCF groups. (**C**–**F**) Fecal concentrations of acetic acid, propionic acid, butyric acid, and total SCFAs in the four groups. Data are presented as mean ± standard deviation (*n* = 5). * *p* < 0.05, ** *p* < 0.01, *** *p* < 0.001, ns: no significant difference vs. the SD group. Statistical analysis was performed using one-way ANOVA.

To determine changes in gut microbiota at the genus level, the top 15 gut microbes were listed, with *Muribaculaceae_unclassified*, *Lactobacillus*, *Desulfovibrio*, and *Ligilactobacillus* as dominant genera (Figure 4A). Compared with the CT group, SD significantly increased the relative abundances of *Desulfovibrio*, *Mucispirillum*, and *Staphylococcus* (*p* < 0.05) and decreased those of *Lactobacillus* and *Paramuribaculum* (*p* < 0.01). Notably, FCF treatment reversed the changes caused by SD. In the FCF-treated group, the relative abundances of *Muribaculaceae_unclassified*, *Lactobacillus*, and *Akkermansia* were significantly increased (*p* < 0.05), while those of *Staphylococcus* (*p* < 0.01) and *Desulfovibrio* (*p* < 0.01) were significantly decreased (Figure 4B). Notably, FCF treatment significantly increased the proportions of beneficial bacteria such as *Lactobacillus* and *Akkermansia*.

Subsequently, SCFAs concentrations were measured to explore the potential physiological roles of gut microbiota metabolites in this process. SD treatment significantly reduced total fecal SCFAs concentration (*p* < 0.001) (Figure 4F), including acetic acid (*p* < 0.01), propionic acid (*p* < 0.05), and butyric acid (*p* < 0.01) (Figure 4C–E), indicating that SD disrupted SCFAs production. FCF treatment promoted the production of acetic acid, propionic acid, and butyric acid, particularly fecal butyric acid concentration (*p* < 0.01). Additionally, FCF treatment nearly restored butyric acid levels to those of the CT group. Meanwhile, MT treatment also exerted a certain effect on SCFAs production: compared with the SD group, it significantly increased acetic acid, propionic acid, and total SCFAs (*p* < 0.05), though its efficacy was slightly inferior to that of FCF. Collectively, FCF promoted fecal SCFAs production, especially butyric acid concentration, in SD mice.

### 3.5. FCF Alleviates SD-Induced Colon Tissue Damage and Intestinal Inflammation

**Figure 5:**
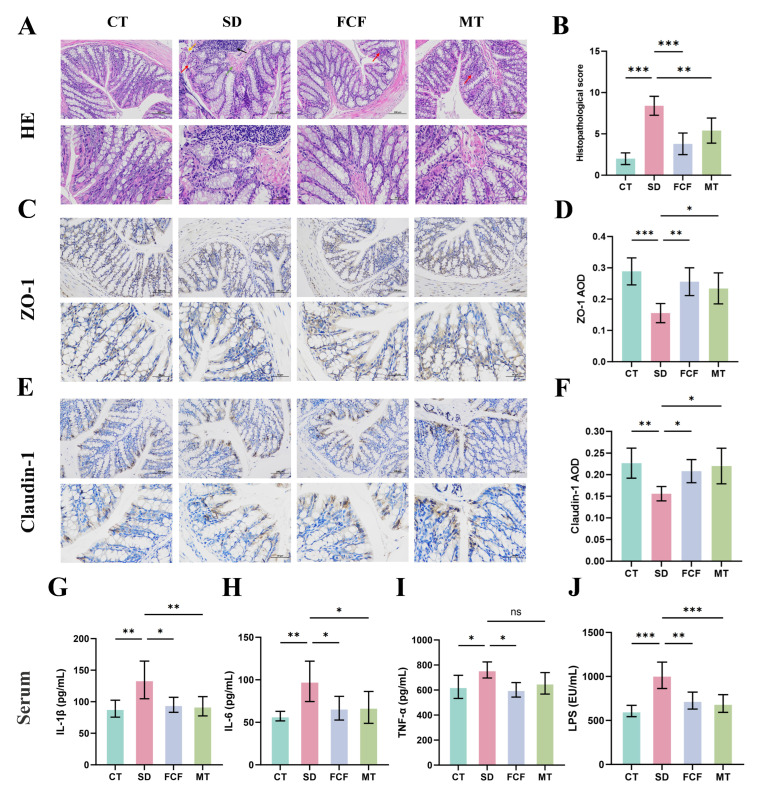
Effects of FCF on intestinal barrier function in sleep-deprived mice. (**A**) Representative H&E staining of colonic tissues. (**B**) Histopathological scoring. (**C**) Immunohistochemical staining of ZO-1. (**D**) AOD of ZO-1. (**E**) Immunohistochemical staining of Claudin-1. (**F**) AOD of Claudin-1. (**G**–**I**) Serum levels of proinflammatory cytokines IL-6, IL-1β, and TNF-α. (**J**) Serum LPS levels. Data are presented as mean ± standard deviation (*n* = 5). * *p* < 0.05, ** *p* < 0.01, *** *p* < 0.001, ns: no significant difference vs. the SD group. Statistical analysis was performed using one-way ANOVA.

These results indicate that gut homeostasis may play a critical role in the recovery of SD-induced cognitive function. Therefore, further investigations were conducted on the effects of FCF intake on gut barrier dysfunction and serum inflammation in SD mice. H&E staining (Figure 5A) showed that compared with the CT group, the SD group exhibited pathological changes such as vascular dilation and hemorrhage, muscular layer thickening, edema in the colonic mucosa and submucosa (green arrows), depletion of goblet cells (red arrows), significantly increased numbers of infiltrating lymphocytes (black arrows), mucosal epithelial cell exfoliation, and irregular crypt surfaces (yellow arrows). Histopathological scoring, based on the colon damage degree criteria (Table S1), showed that SD induced significant intestinal histopathological damage (*p* < 0.001). Notably, both MT and FCF treatment significantly improved SD-induced intestinal histological damage (*p* < 0.01, *p* < 0.001) (Figure 5B).

Additionally, to determine changes in gut barrier function, immunohistochemical staining was used to detect the expression of tight junction proteins ZO-1 and Claudin-1, essential for the intestinal barrier (Figure 5C,E). Results showed that the expression of ZO-1 and Claudin-1 in the model (SD) group was significantly decreased (*p* < 0.001, *p* < 0.01). FCF treatment increased the expression of ZO-1 and Claudin-1 (*p* < 0.01, *p* < 0.05) (Figure 5D,F), with effects on par with MT treatment.

When gut barrier function is impaired, LPS can exacerbate inflammation, disrupt the epithelial barrier, and increase intestinal permeability. To further confirm this damage, LPS levels in serum were measured. Compared with the CT group, LPS levels in the SD group were significantly increased (*p* < 0.001), whereas both FCF and MT treatments significantly reduced LPS levels compared with the SD group (*p* < 0.01, *p* < 0.001) (Figure 5J).

Reduced expression of SCFAs and tight junction proteins can lead to disruption of intestinal epithelial barrier permeability and altered formation of inflammatory cytokines. Therefore, proinflammatory cytokines in serum, including IL-6, IL-1β, and TNF-α, were further analyzed. SD significantly increased the concentrations of IL-1β, IL-6, and TNF-α (*p* < 0.01, *p* < 0.01, *p* < 0.05) (Figure 5G–I). Collectively, SD-treated mice exhibited intestinal inflammation, leading to weakened gut barrier function, allowing proinflammatory cytokines to enter the peripheral circulation and induce systemic inflammation. However, FCF significantly alleviated this inflammatory state (*p* < 0.05), with an improvement effect on par with the MT group.

### 3.6. Effects of FCF on Activation of Hippocampal Glial Cells and TLR4/NF-κB Signaling Pathway

**Figure 6:**
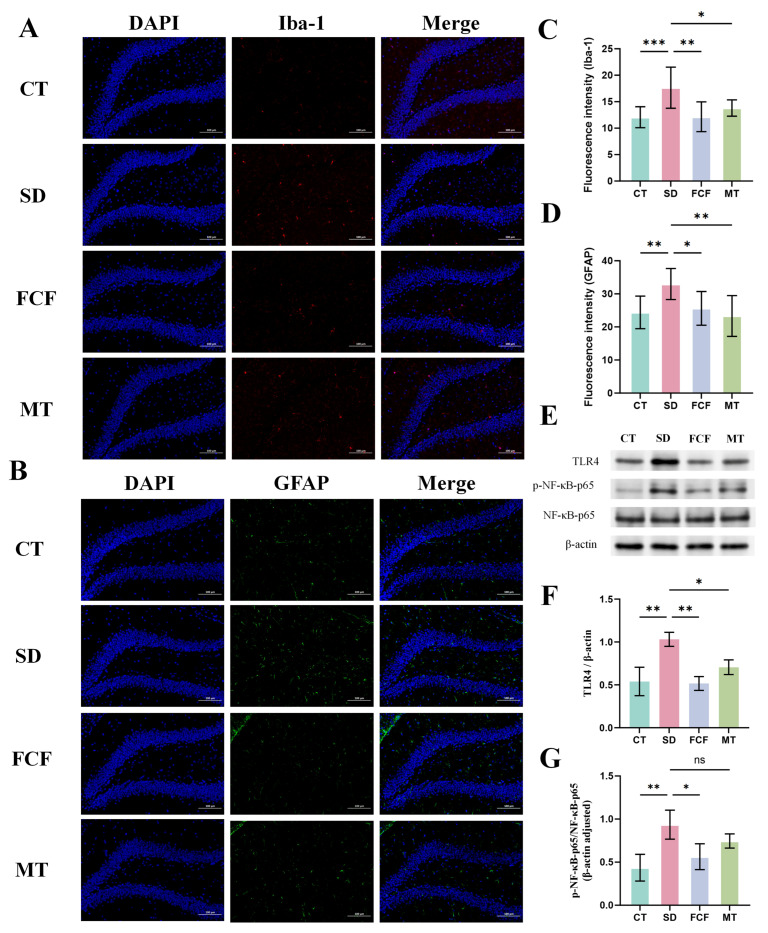
Effects of FCF on hippocampal glial cell activation and TLR4/NF-κB signaling pathway in sleep-deprived mice. (**A**) Representative immunofluorescence staining images of Iba1 in the hippocampal DG region. (**B**) Representative immunofluorescence staining images of GFAP in the hippocampal DG region. (**C**) Fluorescence intensity of hippocampal Iba-1. (**D**) Fluorescence intensity of hippocampal GFAP. (**E**) Levels of TLR4, NF-κB p65, and p-NF-κB p65 in the hippocampus were determined by Western blotting. (**F**) Relative protein levels of TLR4. (**G**) Ratio of p-NF-κB p65 to total NF-κB p65. Data are presented as mean ± standard deviation (*n* = 3–5). * *p* < 0.05, ** *p* < 0.01, *** *p* < 0.001, ns: no significant difference vs. the SD group. Statistical analysis was performed using one-way ANOVA.

Peripheral LPS activates microglia and astrocytes, inducing CNS neuroinflammation, and their collaboration in neuroinflammation leads to neuronal death and synaptic loss. Expression levels of Iba-1 and GFAP in microglia and astrocytes were assessed via immunofluorescence labeling in the hippocampus (Figure 6A,B). Results showed upregulated Iba-1 and GFAP expression in microglia and astrocytes within the hippocampal dentate gyrus (DG) region of model mice (*p* < 0.001, *p* < 0.01) (Figure 6C,D). However, both Iba-1 and GFAP levels were reduced following FCF treatment (*p* < 0.01, *p* < 0.05). FCF showed an overall performance comparable to that of the MT group, but the reduction in Iba-1 expression in the FCF group was greater than that in the MT group, while GFAP expression was slightly higher.

TLR4 receptors on glial cells respond to LPS and harmful substances. Thus, further investigations were conducted to determine whether FCF alleviates neuroinflammation by regulating LPS-induced TLR4/NF-κB p65 pathway activation. Levels of TLR4, NF-κB p65, and p-NF-κB p65 in hippocampal tissues were detected via Western blot (Figure 6E). Results showed significantly elevated TLR4 and p-NF-κB p65/NF-κB p65 levels in the SD group compared to the CT group (*p* < 0.01) (Figure 6F,G), indicating activation of the TLR4/NF-κB signaling pathway by SD. In contrast, FCF treatment first reduced TLR4 expression (*p* < 0.01) (Figure 6F) and then decreased the subsequent p-NF-κB p65/NF-κB p65 level (*p* < 0.05) (Figure 6G), with an overall effect superior to that of the MT group.

### 3.7. FCF Protects Hippocampal Neuronal Cells and Regulates Neurotransmitter Metabolism

**Figure 7:**
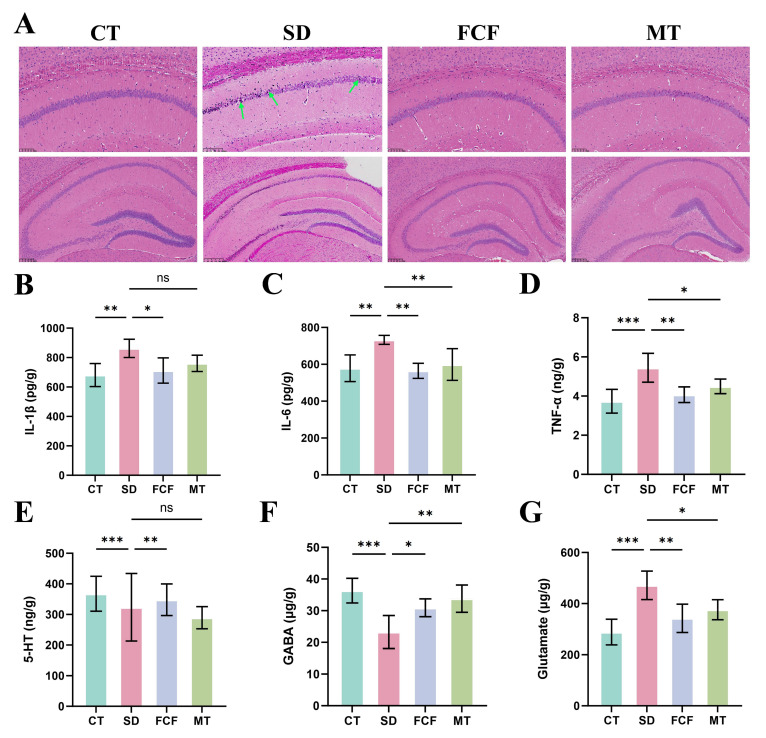
Effects of FCF on neuroinflammation and neurotransmitters in sleep-deprived mice. (**A**) Representative images of H&E staining in the mouse hippocampus. (**B**–**D**) Levels of proinflammatory cytokines IL-6, IL-1β, and TNF-α in serum. (**E**) Expression level of 5-HT in the hippocampus. (**F**) GLU level in the hippocampus. (**G**) GABA level in the hippocampus. Data are presented as mean ± standard deviation (*n* = 5). * *p* < 0.05, ** *p* < 0.01, *** *p* < 0.001, ns: no significant difference vs. the SD group. Statistical analysis was performed using one-way ANOVA.

To demonstrate that FCF improves hippocampal symptoms in sleep-deprived mice by reducing glial cell activation and inhibiting the TLR4/NF-κB signaling pathway, various indices in the hippocampus were measured to investigate the effects of FCF on SD-induced cognitive dysfunction. H&E staining (Figure 7A) showed that compared with the CT group, the SD group exhibited obvious pathological changes in the hippocampal CA1 region, including disordered arrangement of neurons, nuclear shrinkage and deep staining (green arrows), disintegration of cellular structures, and disruption of tissue morphology. After FCF treatment, the arrangement of neurons in the hippocampal CA1 region became more ordered, with improved nuclear morphology and chromatin distribution.

To examine the improvement effect of FCF on hippocampal neuroinflammation in SD mice, inflammatory cytokines in the hippocampus were measured. Results showed that IL-1β, IL-6, and TNF-α levels in the SD group were significantly higher than those in the CT group. Compared with the SD group, FCF treatment significantly reduced IL-1β (*p* < 0.05), IL-6 (*p* < 0.01), and TNF-α (*p* < 0.01) levels in SD mice (Figure 7B–D).

By detecting neurotransmitter expression in the hippocampus of mice, SD caused decreased levels of 5-HT and GABA, alongside a significant elevation in GLU levels (*p* < 0.001) in mouse brains, whereas FCF treatment significantly rectified these abnormalities (Figure 7E–G). Specifically, following FCF treatment, 5-HT and GABA concentrations increased significantly (*p* < 0.01), GLU levels were notably reduced (*p* < 0.01), and the GABA/GLU ratio was restored to near-normal levels. Compared with the MT group, FCF treatment exhibited a more pronounced improvement in 5-HT levels, while showing comparable effects on GABA and GLU levels.

## 4. Discussion

Sleep deprivation (SD), a prevalent health concern in modern society, is strongly linked to cognitive deficits tied to CNS inflammation and gut microecological imbalance [20]. Notably, SD severely impairs the acquisition and consolidation of learning and memory, while also inducing mental health conditions such as anxiety and depression, which seriously disrupt work and daily life [36]. SD impairs hippocampus-dependent cognitive functions spatial memory and recognition memory, and induces anxiety-like behaviors consistent with prior findings that SD triggers cognitive dysfunction [37,38]. These deficits are significantly alleviated by FCF, as evidenced by improved performance in behavioral tests.

Experimental findings revealed marked dysbiosis of the gut microbiota at the phylum level in SD mice: the *Firmicutes*/*Bacteroidota* (F/B) ratio is significantly elevated, a change typically linked to abnormal intestinal energy metabolism and inflammatory states [39]. Overproliferation of *Firmicutes* may exacerbate metabolic disorders by enhancing host absorption of dietary fat, while reduced abundance of *Bacteroidota* directly weakens their capacity to produce SCFAs and maintain intestinal mucosal barrier integrity, thereby facilitating pathogenic bacteria adhesion [40]. Meanwhile, the abundance of *Actinobacteria* decreased. As an important symbiotic phylum, its members, such as *Bifidobacteria*, can inhibit the NF-κB signaling pathway by secreting anti-inflammatory cytokines and regulating the expression of tight junction proteins. A decrease in its abundance may further aggravate intestinal mucosal damage induced by SD [41]. After FCF intervention, the phylum-level microbial structure is significantly reversed: *Firmicutes* proportion decreases, and *Bacteroidota* and *Actinobacteriota* abundances rebound. This suggests that FCF may improve the intestinal barrier and reduce activation of inflammatory pathways by altering the abundances of these microbial phyla.

At the genus level, SD mice exhibited significantly reduced abundances of beneficial bacteria, including *Lactobacillus* and *Akkermansia*. As a core genus producing lactic acid and SCFAs, the reduction in *Lactobacillus* leads to an increase in intestinal pH, facilitating pathogenic bacteria proliferation, while weakening its barrier-protective effect of promoting ZO-1 protein expression through activating the TLR2 pathway [42]. A reduction in *Akkermansia* may impair the integrity of the intestinal mucosal mucus layer. This bacterium sustains the thickness of the mucus layer by degrading mucin, restricting LPS translocation through upregulating the tight junction protein occludin; notably, its decreased abundance correlates positively with elevated serum LPS levels in SD mice [43]. Conversely, pathogenic bacteria such as *Desulfovibrio* and *Staphylococcus* are enriched. The former damages the mitochondrial membrane potential of intestinal epithelial cells by metabolizing hydrogen sulfide, while certain types of the latter may disrupt the intestinal mucosal barrier by secreting toxins such as α-hemolysin [44]. Their synergistic effect can activate TLR4 receptors on intestinal mucosal immune cells, inducing the release of proinflammatory cytokines such as IL-6 and TNF-α [44,45]. After FCF intervention, the abundances of *Lactobacillus*, *Akkermansia*, and *Muribaculaceae_unclassified* are significantly upregulated, suggesting that FCF may thereby repair the intestinal mucosal barrier, inhibit intestinal inflammatory responses, and potentially improve cognitive function [46]. Meanwhile, the abundances of *Desulfovibrio* and *Staphylococcus* are significantly suppressed, indirectly diminishing the production of proinflammatory metabolites, consistent with previous studies showing that flavonoids alleviate neuroinflammation by inhibiting intestinal pathogenic bacteria [47]. Currently, numerous studies are exploring substances that improve sleep deprivation-induced gut microbiota dysbiosis. For example, studies on Fructus gardeniae have shown that it ameliorates gut microbiota dysbiosis in sleep-deprived rats by increasing the abundance of beneficial bacteria like *Muribaculaceae* and *Lactobacillus* and decreasing the abundance of *Lachnospiraceae_NK4A136_group*, thereby alleviating anxiety-like behaviors [48]. Similar to FCF, Fructus gardeniae exerts its effects through regulating gut microbiota, though it targets different specific microbial taxa, reflecting the diversity of microbiota-modulating strategies in counteracting sleep deprivation-related disorders.

SCFAs, core metabolic signaling molecules produced by gut microbiota via the microbiota–gut–brain axis and mainly including butyric acid, propionic acid, and acetic acid, are generated by colonic microbiota via fermenting dietary polyphenols. They provide energy for the intestinal epithelium and regulate central nervous functions through blood circulation and the BBB [49,50]. In SD mice, fecal acetic acid, propionic acid, and butyric acid levels are significantly reduced. FCF intervention increased the abundance of SCFAs-producing beneficial bacteria, including *Lactobacillus*, *Akkermansia*, and *Lachnospiraceae*, restoring acetic acid and propionic acid levels and bringing butyric acid concentration to normal levels. Butyric acid, the only SCFA capable of crossing the BBB, has been verified to inhibit glial cell activation, reduce proinflammatory cytokine levels in the hippocampus, and enhance BDNF to improve mitochondrial function and synaptic plasticity [51]. Propionic acid inhibits microglial activation and hippocampal oxidative stress and interferes with Aβ neurotoxic oligomer formation [52]. At low concentrations, acetic acid enhances the intestinal barrier to reduce LPS translocation into the bloodstream, while at high concentrations, it may inhibit microglial phagocytosis of Aβ plaques. FCF prevents its abnormal increase by regulating microbiota balance [53]. Mechanistically, FCF may target and regulate gut microbiota structure, restore the overall balance of SCFAs, and enhance intestinal barrier integrity to reduce systemic inflammation.

Cognitive impairment is linked to gut microbiota dysbiosis, while the intestinal mucosal barrier is tightly associated with gut homeostasis. Acting as a physical, chemical, immune, and biological barrier, the intestinal mucosal barrier serves as the first line of defense separating luminal contents from host tissues [54]. Prior research has demonstrated that sleep deprivation (SD) can induce impairment of colonic barrier function and enhanced susceptibility to pathogenic organisms, findings that align with the outcomes of the present study [55]. The homeostasis of the intestinal epithelial barrier is regulated by tight junction proteins [56]. Further measurements showed that FCF increased the expression of intestinal epithelial tight junction proteins ZO-1 and Claudin-1 in SD mice, suggesting that FCF can improve the damaged mucosal barrier and restore epithelial integrity in SD mice. Numerous studies have focused on identifying substances that improve intestinal barrier function in sleep-deprived mice. For instance, studies have shown that sleep deprivation induces small intestinal mucosal injury, and melatonin supplementation alleviates such injury by inhibiting oxidative stress and inflammation [57]. There are commonalities between this finding and our study: both melatonin and FCF exert protective effects by improving intestinal barrier integrity and mitigating SD-induced inflammatory responses. Notably, in our study, a melatonin group was also included, and its efficacy in ameliorating SD-induced intestinal barrier impairment was comparable to that of the FCF group, further supporting the shared protective potential of these interventions in counteracting SD-related intestinal damage. Previous research has demonstrated that *Akkermansia* can increase the expression of tight junction proteins, ultimately improving intestinal barrier integrity, which may be one of the pathways for FCF’s beneficial effects [58]. Notably, SD-induced intestinal barrier disruption leads to LPS translocation into the bloodstream, triggering systemic inflammatory responses and increasing serum levels of proinflammatory cytokines. FCF supplementation improves intestinal mucosal integrity, thereby alleviating this condition.

Glial cells have gained increasing attention in biological and physiological studies of CNS diseases. As the primary immune cells in the CNS, microglia maintain CNS homeostasis by phagocytosing foreign substances, clearing dead cells, and releasing cytokines. In their resting state, microglia exhibit a ramified morphology, facilitating monitoring of the neuronal microenvironment and regulation of synaptic pruning, plasticity, and neuronal activity. Upon sensing neuronal injury or inflammatory signals, they rapidly activate, transform into an amoeboid morphology, and secrete abundant proinflammatory cytokines. Microglia can polarize into proinflammatory M1 and anti-inflammatory M2 phenotypes: the former exacerbates neurotoxicity by releasing GLU, while the latter exerts neuroprotective effects by secreting IL-10 and other cytokines [59]. Astrocytes participate in neural homeostasis by regulating the BBB, clearing GLU from synaptic clefts, buffering potassium ions, and secreting neurotrophic factors. Under inflammatory conditions, they can transition from a neuroprotective A2 phenotype to a neurotoxic A1 phenotype, exacerbating neuronal damage by secreting complement components and other factors [60]. Chronic neuroinflammation triggered by excessive microglial activation and the neurotoxic state of astrocytes both accelerate disease progression. M1 polarization of microglia enhances pathological protein deposition, while the A1 state of astrocytes accelerates neuronal loss through inflammatory cascades [61]. Both cell types express transmembrane pattern recognition receptors such as TLR4. LPS can activate the NF-κB pathway by binding to TLR4 on microglia, promoting proinflammatory cytokine expression. Activation of TLR4 in astrocytes accelerates Aβ production and contributes to the pathological processes of Alzheimer’s disease [62,63].

SD impairs intestinal barrier function in mice, leading to increased peripheral LPS levels. As a natural ligand for TLR4, LPS penetrates the BBB through multiple mechanisms: First, LPS induces endothelial cells to produce inflammatory mediators such as TNF-α and IL-1β, directly disrupting the expression and function of BBB tight junction proteins [64]. Additionally, LPS-triggered oxidative stress damages the cytoskeletal structure of endothelial cells via reactive oxygen species, leading to widened intercellular gaps [65]. Once in the brain, LPS binds to TLR4 on microglia and astrocytes in the hippocampus, triggering the MyD88-dependent signaling pathway. This pathway recruits IRAK molecules and activates TAK1, which, in turn, phosphorylates the IκB kinase complex. This process promotes degradation of IκBα and releases NF-κB dimers (p65/p50), ultimately driving nuclear translocation of the p65 subunit and initiating proinflammatory gene transcription [66]. This cascade polarizes microglia from a resting (M0) state to a proinflammatory M1 phenotype, releasing neurotoxic substances like GLU and nitric oxide, while prompting astrocytes to transition from neuroprotective A2 to neurotoxic A1 phenotypes that secrete complement component C3, exacerbating neuronal damage [67]. Immunofluorescence showed upregulated Iba-1 and GFAP in the hippocampal DG of SD mice, confirming excessive glial activation. Western blot showed elevated TLR4 and p-NF-κB p65, validating TLR4/NF-κB pathway activation. FCF upregulated intestinal tight junction proteins ZO-1 and Claudin-1, ameliorated SD-induced intestinal mucosal damage, and restored barrier integrity. This effectively inhibited the translocation of LPS from the intestine to the circulatory system. This repair reduced LPS binding to TLR4 receptors on hippocampal microglia, thereby inhibiting activation of the NF-κB signaling pathway. Notably, FCF inhibited IκB kinase activity, decreased phosphorylation of the NF-κB p65 subunit and its nuclear translocation, thereby downregulating the transcription and secretion of proinflammatory cytokines IL-1β, IL-6, and TNF-α. This regulatory pathway is also supported by other studies. For example, Zhang et al. found quercetin ameliorates SD-induced memory impairment by directly suppressing the TLR4/MyD88/NF-κB pathway to inhibit microglial activation, whereas FCF acts indirectly via intestinal barrier repair to reduce LPS-mediated pathway activation [68].

Detection of various physiological indices in the hippocampus also corroborated the effects of FCF. H&E showed FCF improved SD-induced hippocampal CA1 neuronal abnormalities, and ELISA showed FCF reduced hippocampal inflammatory cytokines to alleviate neuroinflammation. Notably, FCF’s inhibition of glial cells correlated with improved neurotransmitter metabolism. SD decreased brain levels of the inhibitory neurotransmitters 5-HT and GABA, while increasing the excitatory neurotransmitter GLU. FCF intervention increased 5-HT and GABA contents, decreased GLU levels, and restored the GABA/GLU ratio to near-normal. Similar findings have been reported in studies on traditional Chinese medicine interventions. Specifically, studies have demonstrated that Guhan Yangsheng Jing, a traditional Chinese patent medicine, restores neurotransmitter balance of 5-HT, GABA, and GLU, and improves cognitive function. While FCF achieves these effects through microbiota–gut–brain axis regulation and intestinal barrier repair, Guhan Yangsheng Jing directly targets neuronal pathways, highlighting diverse therapeutic strategies for SD-related cognitive impairment [69]. This neurotransmitter balance reestablishment may involve SCFAs regulation. For example, butyric acid can inhibit histone deacetylases by activating G protein-coupled receptors, thereby regulating neuronal gene expression and synaptic plasticity [70]. Meanwhile, SCFAs may indirectly affect the release of brain monoamine neurotransmitters through vagal nerve pathways [71]. These mechanisms highlight the multitarget cooperative action profile of FCF.

Recent studies in this field have primarily focused on how bioactive compounds influence cognitive ability and various brain indices in SD mice [72,73,74]. However, most have not explored the comprehensive mechanisms underlying these effects or the contribution of the microbiota–gut–brain axis. Our previous study used a constant darkness-induced circadian rhythm disorder model, focusing on FCF’s regulation of intestinal flora, barrier function, and hippocampal Aβ and inflammatory factors [7]. In contrast, this study employs a sleep-deprived mouse model and further explores in-depth how FCF treatment modulates intestinal barrier function and gut microbiota, which in turn ameliorates hippocampal TLR4/NF-κB pathway activation and glial cell overactivation via the microbiota–gut–brain axis, thereby alleviating neuroinflammation and mitigating cognitive impairment in SD mice. Compared with our previous study, this study further delves into the specific mechanisms by which FCF ameliorates sleep-related cognitive impairment. It thereby provides novel insights and strategies for natural bioactive compounds to ameliorate sleep deprivation-related cognitive dysfunction via the microbiota–gut–brain axis.

This study systematically revealed the mechanism by which FCF alleviates SD-induced cognitive impairment through regulation of the microbiota–gut–brain axis, but the study’s limitations must also be acknowledged. For instance, due to interfering factors and the limited number of animals, there were some outliers in the animal behavioral experiments, which cannot fully illustrate FCF’s cognitive improvement efficacy. Additionally, the causal role of specific bacterial species was not clarified via germ-free mice colonization experiments. As a complex mixture of flavonoids, FCF’s gut metabolite profiles, and direct interaction mechanisms with the microbiota remain incompletely understood, and the safety and dose–response relationships of long-term intervention require further validation. At the translational application level, comprehensive dietary strategies can be developed based on the “medicinal and edible substances” framework, and their preventive efficacy against mild cognitive impairment can be verified in sleep-deprived populations. Future research could integrate techniques such as fecal microbiota transplantation, metabolomics, and single-cell transcriptomics to deeply dissect FCF’s action pathways and explore its application potential in SD and other neurodegenerative diseases.

## 5. Conclusions

In summary, the results of this study indicate that chronic SD leads to a series of symptoms in mice, including cognitive impairment, gut microbiota dysbiosis, and excessive activation of glial cells. FCF treatment can reverse SD-induced gut microbiota dysbiosis, restore colonic histopathological changes, improve intestinal barrier function, and reduce the translocation of LPS into serum. Meanwhile, FCF alleviates hippocampal neuronal damage and neuroinflammation by inhibiting the activation of glial cells and downregulating the expression of the TLR4/NF-κB pathway. Additionally, FCF regulates neurotransmitter metabolism in the hippocampus, increasing the levels of 5-HT and GABA while decreasing GLU levels. These findings suggest that FCF exerts neuroprotective effects against SD-induced cognitive impairment by modulating the gut microbiota, inhibiting neuroinflammation, and balancing neurotransmitters, providing novel insights into the improvement of SD-related cognitive dysfunction.

## Figures and Tables

**Table 1:** The contents of the main flavonoids in the FCF extract.

Components	Content (mg/g)
Genistein and Apigenin	226.81 ± 12.74
Luteolin	178.23 ± 10.48
Rutin	155.62 ± 8.19
Cynaroside	63.67 ± 4.92
Isoquercitrin and Hyperoside	18.92 ± 1.81

## Data Availability

The original contributions presented in the study are included in the article. Further inquiries can be directed to the corresponding author.

## Supplementary Materials

The following supporting information can be downloaded at: https://www.mdpi.com/article/10.3390/foods14162888/s1, Figure S1: Representative MRM spectra and chemical structures of flavonoids in FCF; Table S1: Colon pathological analysis damage degree scoring criteria.

## Abbreviations

The following abbreviations are used in this manuscript:

FCF	*Ficus pandurate* var. *angustifolia* W.C. Cheng flavonoids
SD	sleep deprivation
SCFAs	short-chain fatty acids
LPS	lipopolysaccharide
CNS	central nervous system
BBB	blood–brain barrier
TLR4	toll-like receptor 4
IL-1β	interleukin-1β
TNF-α	tumor necrosis factor-α
IL-6	interleukin-6
5-HT	5-hydroxytryptamine
GABA	γ-aminobutyric acid
GLU	glutamate
Iba-1	ionized calcium-binding adapter molecule 1
GFAP	glial fibrillary acidic protein
AOD	average optical density
ZO-1	zonula occludens-1
NF-κB	nuclear factor-kappa B
