# Supplementary material for: Flavonoids from Ficus pandurate var. angustifolia W.C. Cheng Restore Cognitive Impairment and Regulate the Gut Microbiota in Sleep-Deprived Mice
Source: Foods. 2025 Aug 20;14(16):2888. doi: 10.3390/foods14162888 (PMC12385838; doi:10.3390/foods14162888)
Supplement: Supplementary file 1 [file foods-14-02888-s001.zip › foods-3795098-supplementary.pdf]

Supplementary data:

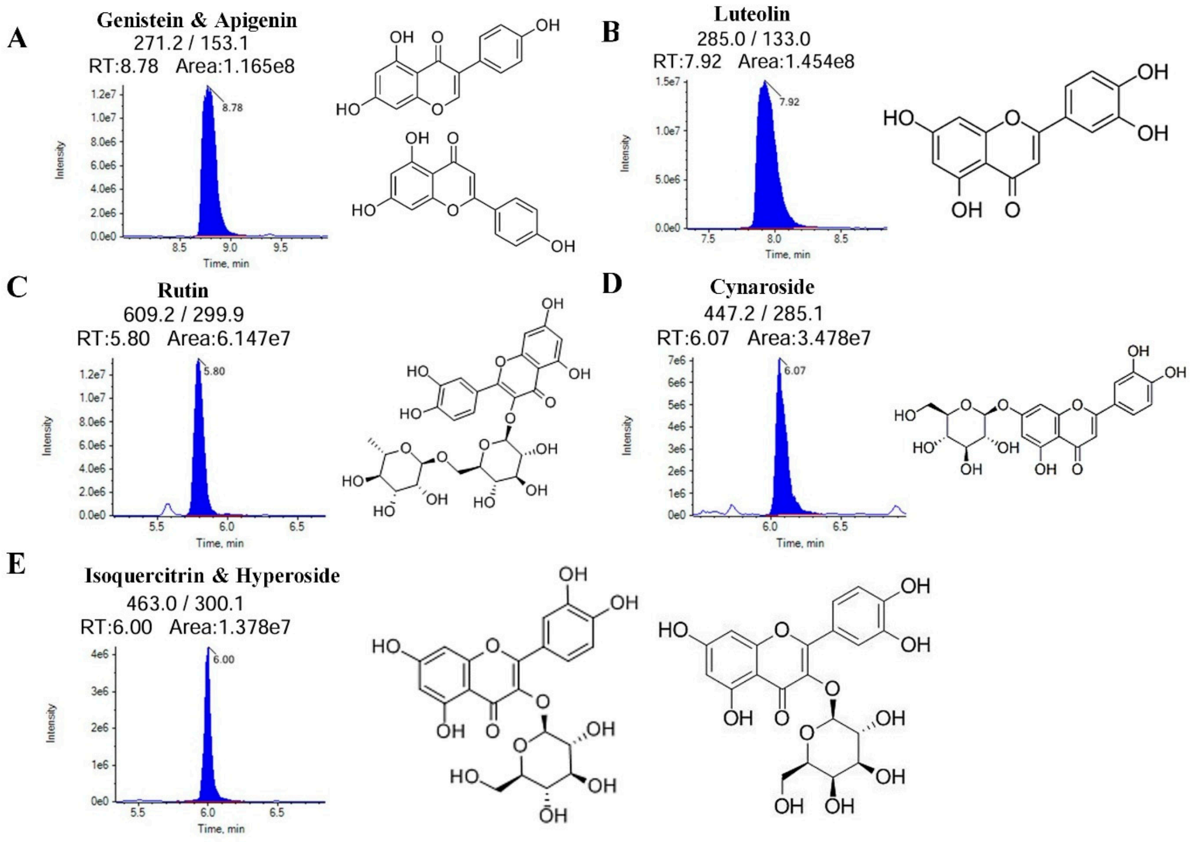

Figure S1. Representative MRM Spectra and Chemical Structures of Flavonoids in FCF.

Table S1. Colon pathological analysis damage degree scoring criteria.

Histological Feature	Score 0	Score 1	Score 2	Score 3	Score 4
**Inflammatory Cell Infiltration**	No infiltration	Mild infiltration	Moderate infiltration	Severe infiltration	-
**Mucosal Damage**	No damage	Damage to mucosal layer	Damage to submucosal layer	Damage to muscular layer	-
**Crypt Destruction**	No destruction	Destruction of 1/3 of crypts	Destruction of 2/3 of crypts	Complete destruction	100% destruction + loss of epithelial cells
**Defect Area**	0%	1-25%	26-50%	51-75%	76-100%
**Epithelial Cell Changes**	Normal	Loss of goblet cells	Extensive loss of goblet cells	Loss of crypts	Extensive loss of crypts or polypoid regeneration
**Hemorrhage**	None	Mild	Moderate	Severe	-
**Submucosal Edema**	None	Mild	Moderate	Severe	-
